# Virtual and Augmented Reality versus Traditional Methods for Teaching Physiotherapy: A Systematic Review

**DOI:** 10.3390/ejihpe12120125

**Published:** 2022-12-02

**Authors:** David Lucena-Anton, Juan Carlos Fernandez-Lopez, Ana I. Pacheco-Serrano, Cristina Garcia-Munoz, Jose A. Moral-Munoz

**Affiliations:** 1 Department of Nursing and Physiotherapy, University of Cadiz, 11009 Cadiz, Spain; 2 Biomedical Research and Innovation Institute of Cadiz (INiBICA), University of Cadiz, 11009 Cadiz, Spain

**Keywords:** augmented reality, higher education, innovation, physiotherapy, teaching, virtual reality

## Abstract

The use of virtual worlds in health-related education is increasingly popular, but an overview of their use in physiotherapy education is still needed. The aim of this review was to analyse the use of virtual and augmented reality (VR/AR) compared to traditional methods for teaching physiotherapy. A systematic review was performed up to October 2022 in PubMed, Web of Science, Scopus, CINAHL, and PsycInfo. The quality appraisal and risk of bias were assessed by the Joana Briggs Institute checklist and the Cochrane Collaboration’s RoB Tool 2.0, respectively. A total of seven randomised and non-randomised controlled studies were included, involving 737 students. VR/AR-based teaching approaches included simulation and virtual worlds, and were conducted through immersive head-mounted displays, AR-based applications, and 3D visualisations. Three studies were focused on teaching anatomy content, two on clinical decision making skills, and the rest were focused on pathology, physiotherapy tasks or exercise performance, and movement analysis of lower limbs. Inconclusive results were found in terms of learning satisfaction and academic performance, showing VR/AR-based teaching models to be equally effective as traditional methods for teaching physiotherapy. We encourage researchers and teachers to include games in their VR/AR-based teaching approaches to enhance interaction and active learning in physiotherapy education.

## 1. Introduction

### 1.1. Information and Communication Technologies in Education

The use of information and communication technologies (ICT) is experiencing exponential growth because of the expansion of using mobile applications and smartphone access [1]. ICT includes a broad range of communication devices, applications, and services, such as social media networks and platforms, audio-visual platforms, internet messaging, and videoconference applications, among others [2].

Concerning education, new and modern trends are being adopted by educators [2], and traditional teaching and learning models based on lectures are being complemented, and even replaced, by teaching models based on ICT [3,4]. In this line, Delgado et al. [3] emphasised awakening values in students that allow them to use educational resources and media (ICT) responsibly and optimally for the acquisition of knowledge, in addition to the following basic principles for teaching: to encourage active learning, to provide timely feedback, to promote contact between students and teachers, to encourage cooperation between students, to promote the appropriate use of time, to encourage student’s high expectations, and to respect diverse learning styles. These ICT-based teaching models are considered an innovative tool that can improve motivation and stimulation for student’s learning, serving as a complementary educational resource for the use of traditional teaching models [5,6]. This growth in the use of ICT in education also took place in the field of health-related education, in which technology could help to improve the educational experience by increasing understanding and psychomotor skills [7]. Finally, regarding adult education, the inclusion of ICT allows the creation and dissemination of digital media literacy to improve knowledge acquisition and to develop the 4C skills: collaboration, communication, critical thinking, and creativity [8].

### 1.2. Virtual and Augmented Reality

Virtual reality (VR) is defined as the “*use of interactive simulations created with computer hardware and software to present users with opportunities to engage in environments that appear and feel similar to real-world objects and events*” [9]. This technology allows us to explore and manipulate the content of virtual environments in real-time [10]. In addition, the content of the virtual environments can be created for specific purposes, and it can be manipulated in terms of duration, intensity, and feedback [11].

VR systems include a wide number of devices which can be divided according to the user immersion according to two groups [12]: (i) immersive systems: users are totally integrated into the virtual environment; and (ii) semi-immersive systems: users are partially integrated into the virtual environment. Head-mounted displays and caves are examples of VR immersive devices, whilst computer screens displaying the environment are examples of VR semi-immersive devices. It the difference between VR and augmented reality (AR) should be addressed. VR comprises involving the user into the virtual environment, and AR displays virtual images in the real physical environment [13].

The use of virtual learning environments provides several advantages, such as the opportunity to use a motivating context [14], the sustained focus of attention on the task performed, as well as the opportunity to provide feedback continuously. All these aspects can lead to the higher achievement of the objectives in the learning process [15].

### 1.3. Virtual and Augmented Reality for Health-Related Adult Education

Scientific literature stated that the incorporation of new and innovative teaching models based on the use of virtual learning environments can favour learning stimulation on students, largely due to the use of a playful, fun, and easy-to-use context [16]. The inclusion of educational resources based on the use of video games in the teaching methodology also proved to be useful in increasing student satisfaction with the teaching process [17]. The potential inclusion of VR/AR systems in education is emerging among researchers [18], since it could allow students to explore a broad range of scenarios that are even impossible to set up in the traditional classroom. It also provides a multi-sensory interactive medium promoting similar behaviours in learning to those in the real world [19]. Other benefits of using VR/AR in education were shown by several studies performed across different disciplines: Cesar Ferreria et al. [20], found that students shared information, asked questions, and moved towards the correct answer when solving physics problems in virtual environments, showing assertive and argumentative discussions; Vidal et al. [21] found high satisfaction rates among students after using AR for pedagogy learning. Moreover, students considered that AR could be useful for conflict resolution, and it offers multiple application possibilities and functional feasibility to make their projects; Dyer et al. [22] found that VR was an effective method to teach empathy among medical and health professional students which showed also a higher understanding of age-related health problems; and Shorey and Debby Ng [23] suggested that VR simulation was effective to improve learning outcomes, such as theoretical knowledge, and it could be used as an alternative or complement to teaching in nursing education. In spite of the above findings, Berns et al. [24], who performed a recent review analysing the use of VR apps obtained from commercial platforms for language learning, highlighted that the current VR apps did not use interactive environments in which interaction was similar to real-world interaction, so the sense of immersion and, consequently, the learning impact could be limited. These authors also suggested that future apps should use learning approaches such as explorative, experiential, constructive, and collaborative learning, and a user-centred design approach should be implemented for creating new apps.

Finally, according to Merchant et al. [15] and Rojas-Sánchez et al. [18], the success of using VR/AR for teaching purposes could depend on the selected instructional design principles, such as games, simulation, or virtual worlds, with games having the greatest effect on learning due to the interactive participation, rather than passively receiving the information to be learned.

### 1.4. New Technologies for Teaching Physiotherapy

Regarding physiotherapy education, students should acquire the essential theoretical knowledge about anatomy, physiology, kinetics, kinematics, etc. [25]. In addition, different specific skills should be learned before starting their professional career in different healthcare settings. These specific skills should include observation, examination [25], specific tasks or exercises, and clinical decision-making skills [26], and their acquisition requires students to integrate multiple complex factors, hypothesis formulation, problem solving, etc., which may be difficult to acquire through traditional lectures, including lectures and case study demonstrations, tutorials, practical classes, etc. [27]. In this line, it was shown that using audio–visual material can lead to improvements in terms of learning interest by physiotherapy students, as well as improvements in the acquisition of theoretical content and practical skills [26].

### 1.5. Justification and Objective

Although the use of VR/AR in other education fields was studied, to the best of our knowledge, there is a lack of systematic review analysing the use of VR/AR for teaching physiotherapy compared to traditional methods, so an overview of the use of new teaching tools based on VR/AR among physiotherapy education is still needed. Because of the specific special skills and theoretical knowledge to be taught during physiotherapy teaching, we hypothesise that using VR/AR as a teaching tool could be more useful than traditional methods for teaching physiotherapy due to its advantages in terms of multisensory stimulation, interaction, playful environment, and feedback. Therefore, the objective of the present systematic review is to assess the scientific evidence of using VR/AR compared to traditional methods for teaching curricular content in physiotherapy among graduate and undergraduate students, providing detailed information about the current use of these tools, and its results on the level of satisfaction and knowledge acquisition.

## 2. Materials and Methods

### 2.1. Design

This systematic review was performed according to the preferred reporting items for systematic reviews and meta-analyses (PRISMA) 2020 recommendations for systematic reviews and meta-analyses [28].

### 2.2. Search Strategy

A search of the scientific literature was carried out in the following databases: PubMed, Web of Science, Scopus, CINAHL, and PsycInfo. The “Grey literature” was not consulted. The search was carried out up to October 2022. The following keywords were used for the searches (Table 1): (“physical therapy” OR “physiotherapy”) AND (“teaching” OR “learning”) AND (“virtual reality” OR “augmented reality” OR “mixed reality” OR “virtual reality exposure therapy” OR “virtual system”). No filters were applied in terms of date of publication or study design.

### 2.3. Eligibility Criteria

The inclusion criteria for the selection of the articles included in this review were as follows: the study population was made up of graduate or undergraduate students of physiotherapy; the teaching method was carried out using VR/AR-based devices and was compared to traditional teaching methods; the results are related to the learning satisfaction/perception with the teaching model, and the academic performance regarding the acquisition of theoretical knowledge and/or physiotherapy-related skills. Concerning the study types, included studies were randomised and non-randomised controlled studies, written in the English or Spanish language, and published in peer-reviewed journals and conference proceedings.

Regarding the exclusion criteria, we excluded those studies that included a population of physiotherapy students but did not provide detailed and separate results from the other study populations. In addition, studies focused on the acquisition of professional skills were excluded, i.e., those that take professional trainees as the study population.

### 2.4. Selection Process and Data Extraction

First, the search was performed by combining the keywords previously described in the different databases. Potentially relevant articles were identified after reading the title and abstract, and duplicate articles were eliminated. Subsequently, a thorough check for compliance with the inclusion criteria was performed to obtain the articles included in the systematic review. Two reviewers (D.L.A. and J.A.M.M.) actively participated in the selection and review process.

Data collection involving the systematic data extracted by two independent reviewers (D.L.A. and J.A.M.M.) was manually conducted according to the Joana Briggs Institute (JBI) data extraction form [29]. The following information was extracted from each article included in the systematic review: author, year of publication, country, study design, characteristics of the participants, characteristics of the teaching model, number of lessons received, instruments used to assess the impact produced by the teaching model, and results obtained. The synthesis of the information was visually displayed as a table. In case of discrepancies, two additional reviewers (J.C.F.L. and A.I.P.S.) were consulted.

### 2.5. Methodological Quality and Risk of Bias Assessment

The JBI Critical Appraisal Checklist for Quasi-Experimental Studies (ranged from 0 to 9), the JBI Checklist for Cohort Studies (ranged from 0 to 11), and the JBI Checklist for Randomised Controlled Trials (ranged from 0 to 13) [30] were used for conducting the quality appraisal. In addition, the Cochrane Collaboration’s risk of bias tool (RoB 2.0) [31] and the risk of bias in non-randomised studies of interventions (ROBINS-I) tool [32] were used to assess the risk of bias of randomised and non-randomised controlled studies, respectively.

Two reviewers independently (C.G.M. and D.L.A.) conducted the quality appraisal and risk of bias assessment of the retrieved papers. An agreement was reached through discussion, and two additional reviewers (J.A.M.M. and A.I.P.S.) were consulted in case of discrepancies.

## 3. Results

**Figure 1:**
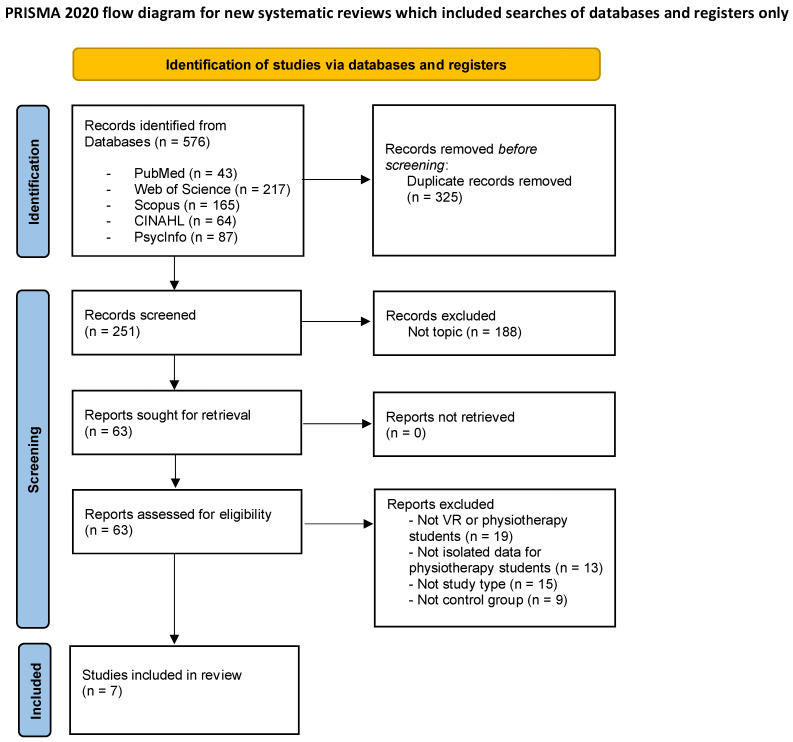
Flow diagram of the selection process for studies in the systematic review.

First, a total of 576 articles were obtained, resulting in 251 articles after removing duplicates. Finally, a total of seven studies were included in the systematic review after verifying the compliance with the pre-established selection criteria. The entire selection process of the articles included in this review is reflected in the following flow chart (Figure 1), which was performed following the PRISMA 2020 recommendations.

The main characteristics of the different studies included in the systematic review are shown in Table 2.

### 3.1. Study Types

In terms of study type, all studies compared VR/AR teaching with traditional teaching models, and five studies [25,33,34,35,38] used randomisation of the participants into different groups. Favolise [36] carried out a cohort longitudinal study, and Kandasamy et al. [37] performed a crossover longitudinal study. From the total number of studies, three of them [25,35,37] used a user-focused product design-oriented research approach.

### 3.2. Results on Methodological Quality and Risk of Bias

After conducting the methodological quality appraisal of randomised controlled studies, some concerns in allocation concealment and blinding of participants and interventions were found. Non-randomised studies reached a JBI score of 2/11 [36], showing a critical risk of bias, and 6/9 [37], showing some concerns. The results obtained on methodological quality assessment through the JBI checklists are shown in Table 2.

**Figure 2:**
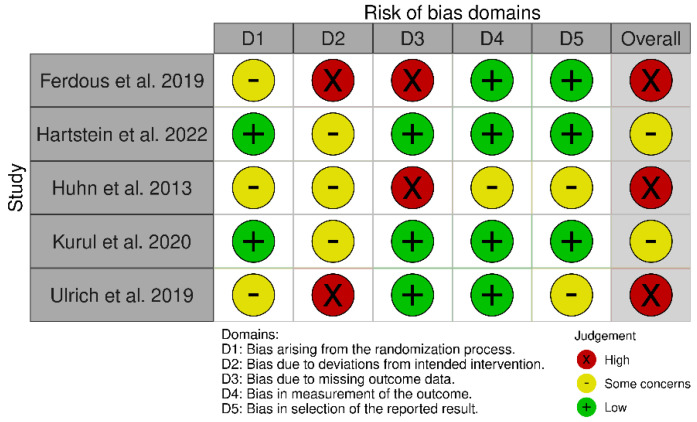
Risk of bias of the randomised controlled studies. Included studies: Ferdous et al. [35], Hartstein et al. [38], Huhn et al. [33], Kurul et al. [25], Ulrich et al. [34].

**Figure 3:**
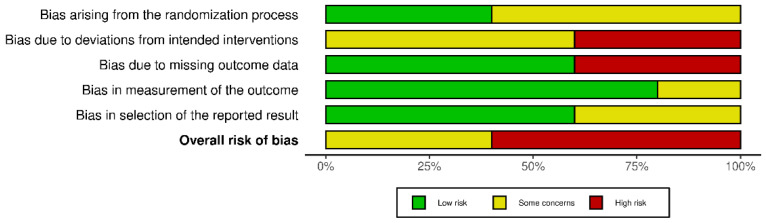
Overall risk of bias for randomised controlled studies. Categories are presented by percentages.

**Figure 4:**
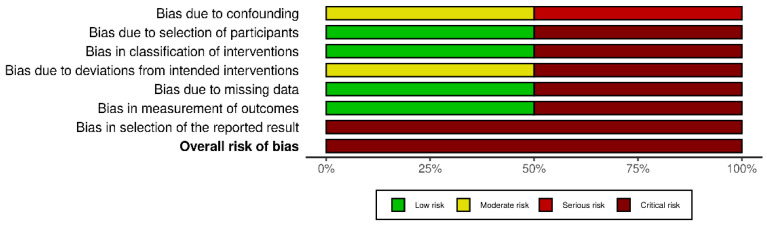
Overall risk of bias for non-randomised studies. Categories are presented by percentages.

Concerning the risk of bias of randomised controlled studies, most studies showed some concerns, while the study by Huhn et al. [33] showed high risk of bias, as shown in Figure 2. The overall score of the RoB 2.0 tool showed high risk of bias due to deviations from the intended intervention and missing outcome data, as shown in Figure 3. Regarding non-randomised studies, an overall critical risk of bias was reached (Figure 4).

### 3.3. Participant Characteristics

The sample sizes included in the studies ranged from 53 subjects [33] to 297 subjects [36]. Thus, the total number of subjects included in the different studies was 737, 105 being the average number of subjects participating across the studies. Graduate students were involved in five studies [33,34,35,36,38], and undergraduate students took part in two studies [25,37].

### 3.4. VR-AR Systems

The VR/AR-based teaching approaches were carried out using immersive VR systems wearing head-mounted displays [25,34,38], AR applications with anatomical projections [35,36,37], and a virtual patient simulator based on computer software [33].

### 3.5. Teaching Content and Duration

Regarding the content of the VR/AR teaching models, there were five types of contents: (i) anatomy, (ii) clinical decision-making skills, (iii) pathology, (iv) physiotherapy tasks or exercise performance, and (v) movement analysis of lower limbs.

First, three studies [25,36,37] were focused on anatomy and palpation content. The study carried out by Kurul et al. [25] compared a teaching model using immersive VR with head-mounted displays to traditional teaching, and the content was related to the anatomy and palpation of the cephalic region and neck, showing statistical differences in favour of the experimental group. Favolise [36] compared AR-based teaching to a traditional method for teaching gross anatomy, and they reported statistical differences in the students’ self-efficacy regarding their ability to learn. Finally, Kandasamy et al. [37] also used an AR-based application for teaching spine anatomy, and they reported statistical differences in the students’ perceived level of understanding and engagement.

Second, two studies [33,38] included content related to clinical decision-making skills. Hartstein et al. [38] used an immersive VR learning experience with the Oculus Quest 2, and they did not find significant results when compared with traditional teaching. The study by Huhn et al. [33] used a virtual patient simulation based on computer software and they found no statistical differences in academic performance and students’ perception of the learning experience.

Third, two studies [33,37] included pathology content in their lessons. The results are contradictory, with Kandasamy et al. [37] obtaining positive results, and Hunh et al. [33] reporting no significant differences.

Fourth, only the study by Ulrich et al. [34] included content related to specific physiotherapy tasks. The authors compared three different models to teach how to perform the correct positioning into the supine position: one based on the projection of 360° videos using VR glasses, another using conventional videos projected on a laptop computer, and another based on traditional teaching. The results are not statistically significant.

Finally, only the study by Ferdous et al. [35] performed a movement analysis of lower limbs in the experimental group, comparing a teaching model based on AR combining the projection of anatomical images with virtual pens to create annotations with a traditional teaching model. The authors reported significant results on academic performance for the AR group.

Concerning the duration of the lessons, the studies performed a limited number of lessons, being only one session in three studies [25,34,38], two sessions in two studies [35,37], and six lessons in the study by Huhn et al. [33]. Favolise [36] did not clearly report the teaching duration.

### 3.6. Study Results

#### 3.6.1. Learning Satisfaction

Regarding the use of ICTs for education purposes, learning satisfaction is related to the effectiveness of the teaching methods to identify the student’s overall satisfaction compared to other teaching situations without technology [34].

Kurul et al. [25], found significant results in students’ perception of the VR experience while learning anatomical structures. Hartstein et al. [38] showed statistical differences between the VR and control group in engagement, which resulted in greater attention during the learning activity, greater enjoyment of their learning opportunity, fewer discouraging emotions, and more frequently entertained new strategies for problem solving. Favolise [36] reported statistical differences in the student’s self-efficacy regarding their ability to learn.

The study by Ulrich et al. [34] showed better results with the VR-based teaching model in the emotional perception of the students, but it was less effective than traditional teaching in students’ learning satisfaction. In this line, Huhn et al. [33] did not show differences in students’ perception of the learning experience.

#### 3.6.2. Academic Performance

Academic performance shows how technology could be effective to measure the student’s performance compared to other situations where technology is not present [34].

Kurul et al. [25] reported significant results for learning anatomical structures through VR. Ferdous et al. [35] reported benefits in understand complex rotational movements of joints. Kandasamy et al. [37] reported statistical differences in the students’ perceived level of understanding of spine anatomy and pathology for the AR group when compared to the traditional teaching group. Favolise [36] reported significant differences in the acquisition of knowledge about cadaver dissection, but not about osteology.

Regarding clinical decision-making skills, the randomised studies conducted by Hartstein et al. [38] and Huhn et al. [33] did not show statistical differences between the experimental and control group. In addition, Hartstein et al. [38] did not reach statistical diagnostic skills, and Huhn et al. [33] did not show differences in knowledge acquisition and transfer of knowledge. Finally, the study by Ulrich et al. [34] showed that VR was equally effective as traditional methods to enhance academic performance.

## 4. Discussion

The present systematic review provides an overview of using VR/AR for teaching physiotherapy among graduate and undergraduate students. To the best of our knowledge, this is the first systematic review assessing this innovative approach in physiotherapy education. A total of seven studies were included in the review. It should be highlighted that a total of 737 students were involved in the analysed studies. After analysing the results of the different studies included in this review, we cannot conclude that using VR/AR as a teaching tool was more effective than traditional teaching methods in terms of learning satisfaction and academic performance, as contradictory results were obtained.

Due to the lack of systematic review or meta-analysis analysing the use of VR/AR for teaching physiotherapy, the present findings will be compared to other systematic reviews and meta-analyses analysing the topic in health-related education. Contrary to our findings, Zhao et al. [39], stated that the pass rate of medical students trained using VR technology was higher than those using traditional education, resulting in a higher acquisition of specialised knowledge. Another recent systematic review carried out by Barteit et al. [40] suggested that using VR/AR teaching models had beneficial effects on medical education, showing positive results on enthusiasm and enjoyment. In addition, Shorey and Debby Ng [23] analysed the use of virtual environments as a teaching tool in nursing education, stating that this teaching model was effective at improving the theoretical knowledge acquisition, even suggesting it as an alternative method to teaching in nursing education. Although our hypothesis was that using VR/AR as a teaching tool would be more useful than traditional methods for teaching specific special skills and theoretical knowledge during physiotherapy teaching, due to its advantages in terms of multisensory stimulation, interaction, playful environment, and feedback, our results are contradictory for both learning satisfaction and academic performance, so no solid conclusion can be drawn about the usefulness of one method over another. In spite of some studies that used immersive VR devices, most studies included in the review used simulations instead of games, which could have less impact on learning due to the lack of interactive participation [15,18]. In this way, according to Ulrich et al. [34], the non-positive results found in this review could be due to the lack of interactivity induction across the different VR/AR-based teaching models used by the studies. In addition, there were heterogeneous factors, such as the teaching content and duration, VR/AR devices used, and academic degree, which could influence the obtained results.

Concerning the teaching content, positive results were found for teaching anatomy (neck, spine, and gross dissection anatomy) [25,36,37], and for acquiring skills related to movement analysis [35], so it could be considered as a useful complement to traditional methods for teaching anatomy content in physiotherapy education. When analysing the results related to the clinical decision-making skills and specific physiotherapy tasks, we found using VR/AR-based teaching methods to be just as effective as traditional methods. A possible explanation for the lack of positive results may be the short teaching period used in the studies, in which improvement trends but non-significant results were observed in the VR/AR group, which could become significant if measured over a greater time period [33]. Therefore, future studies analysing the use of VR/AR to teach these specific skills among physiotherapy students are needed.

Interestingly enough, the positive results obtained for teaching anatomy match with those of previous studies [39,41] analysing the use of VR for teaching anatomy in health-related education. However, we found no positive results for clinical decision-making and specific clinical skills, in contrast to a previous systematic review [42] analysing the use of VR for health profession education. In view of these contradictory results, we can suggest that teaching anatomy may be similar in the different health-related education disciplines, but the specific clinical skills to be developed in each discipline may be different. Finally, considering that there was a limited number of studies analysing each teaching content, the results should be taken with caution.

The overview provided by the present study about the use of VR/AR for teaching physiotherapy could be considered to assess the inclusion of VR/AR in other health-related areas of knowledge. In this way, and according to Zhao et al. [39], we suggest using VR/AR systems as a complement to traditional models instead of replacing traditional models to improve the education of physiotherapy students.

Finally, according to Rickel [43], the inclusion in the university education system of teaching models based on the use of VR would imply an important economic effort for the supply of the infrastructure needed in the different teaching centres, as well as a great effort to offer adequate training and instruction in the use of these new technologies to university teaching staff. In addition, virtual learning environments should be continuously adapted to the needs of teaching staff and students.

### Study Limitations

First, the limited number of studies included, their low methodological quality and high risk of bias, should be highlighted. In addition, several studies used a single teaching lesson, so no strong conclusions can be drawn. Thus, studies implementing VR/AR-based teaching models for improving teaching and learning experience in the medium and long term are needed. In this line, the development of a greater number of experimental studies with higher methodological quality is also needed, as well as studies that encourage the creation of virtual learning environments using user-centred designs, in order to provide a solid conclusion on the use of VR/AR for teaching physiotherapy.

## 5. Conclusions

This systematic review analysed the results of seven studies that examined the use of VR/AR for teaching physiotherapy, with a total of 737 students. Despite the potential benefits of using VR/AR for teaching purposes, such as using a motivating context, the sustained focus of attention on the task performed, as well as the opportunity to provide feedback, which are key factors to induce a higher achievement of the objectives in the learning process, our results are contradictory in terms of learning satisfaction and academic performance. We can conclude that VR/AR-based teaching models seem to be equally effective as traditional methods for teaching physiotherapy. In this way, the scientific evidence of using VR/AR-based teaching methods for teaching physiotherapy is still in its first stages, so we cannot strongly recommend its inclusion in physiotherapy education. In any case, we could recommend it as a complement rather than a replacement for traditional teaching.

We encourage teachers and researchers to conduct future research analysing the use of VR based not only on simulation or virtual environments, but also on games, which could enhance interaction and active learning. In addition, future studies including a larger number of sessions and higher methodological quality are needed to provide solid conclusions on the use of VR/AR for teaching undergraduate and graduate curricular content in physiotherapy.

## Figures and Tables

**Table 1:** Search strategy.

Databases	Keywords	Results
PubMed	(“physical therapy” [Title/Abstract] OR physiotherapy [Title/Abstract]) AND (teaching [Title/Abstract] OR learning [Title/Abstract]) AND (“virtual reality” OR “augmented reality” OR “mixed reality” OR “virtual reality exposure therapy” OR “virtual system”) [Title/Abstract])	43
Web of Science	TS = ((“physical therapy” OR physiotherapy) AND (teaching OR learning) AND (“virtual reality” OR “augmented reality” OR “mixed reality” OR “virtual reality exposure therapy” OR “virtual system”))	217
Scopus	TS = ((“physical therapy” OR physiotherapy) AND (teaching OR learning) AND (“virtual reality” OR “augmented reality” OR “mixed reality” OR “virtual reality exposure therapy” OR “virtual system”))	165
CINAHL	TS = ((“physical therapy” OR physiotherapy) AND (teaching OR learning) AND (“virtual reality” OR “augmented reality” OR “mixed reality” OR “virtual reality exposure therapy” OR “virtual system”))	64
PsycInfo	TS = ((“physical therapy” OR physiotherapy) AND (teaching OR learning) AND (“virtual reality” OR “augmented reality” OR “mixed reality” OR “virtual reality exposure therapy” OR “virtual system”))	87

**Table 2:** Main characteristics of the studies included in the systematic review.

Studies (Authors, Year, Country)	Study Design	Participants	Teaching Models	Duration/Content	Outcomes	Results	Methodological Quality (JBI)
Huhn et al. 2013. USA [33]	Randomised mixed-methods study	N = 53 graduate students (Doctoral) CG: 27 EG: 26	CG: Traditional teaching based on large-group discussion EG: Virtual patient simulation through computer software	Six lessons Content: Pathology content and clinical reasoning	Pre-post measurement Health science reasoning test on clinical reasoning; 50-question exam for knowledge acquisition; Objective clinical structured examination to measure the transfer of learning.	There were no significant differences between groups, but EG showed higher results on all objective measures.	6/13
Ulrich et al. 2019. Denmark [34]	Randomised study	N = 81 graduate students G1: 28 G2: 28 G3: 27	G1: VR HMD with 360° video (Samsung Gear VR) G2: Conventional videos via laptop G3: Traditional teaching	One lesson Content: The practical task of performing the correct positioning into the supine position	Pre-post measurement Questionnaire on academic performance, user satisfaction, and perception of learning climate.	Academic performance: all treatment groups were equally effective. User satisfaction: 360° video and conventional video were less effective than traditional teaching. Learning climate: only in the student’s emotions, the 360° video surpassed the conventional video.	7/13
Ferdous et al. 2019. Australia [35]	Randomised crossover study	N = 101 graduate students G1: 24–26 G2: 24–26 G3: 24–26 G4: 24–26	CG: Traditional teaching EG: AR and projection of anatomical images, virtual pencils to create annotations	Two lessons of 1 h Content: different types of movements of the lower limb musculature	Pre-post measurement Standardised questionnaire type test score.	The results show a statistically significant mean increase in the questionnaire score (22.5%) in the EG with respect to the CG. Z-2.666, *p*-0.008.	7/13
Kurul et al. 2020. Turkey [25]	Randomised controlled study	N = 72 undergraduate students CG: 36 EG: 36	CG: Traditional teaching EG: Immersive VR HMD (Oculus Rift) and “3D Organon Anatomy” software	One lesson of 30 min Content: anatomy and palpation of the cephalic region and neck	Pre-post measurement: Quiz-type questionnaire on anatomy with 15 multiple-choice questions. Likert-type scale on student perception.	Post scores were significantly higher compared to pre-test scores in both EG (*p* < 0.001) and CG (*p* < 0.001). The difference between pretest and post-test scores was significantly greater in favour of EG (*p* < 0.001).	10/13
Favolise 2021. USA [36]	Cohort longitudinal study	N = 297 graduate students (Doctoral) CG: 162 EG: 135	CG: Traditional teaching EG: Visible Body through VR and AR software	N/A Content: Gross anatomy	Post measurement Exams for knowledge acquisition on osteology and cadaver dissection. Survey about self-efficacy.	Positive results were found for the EG group on knowledge acquisition of cadaver dissection, and student’s self-efficacy.	2/11
Kandasamy et al. 2021. United Kingdom [37]	Crossover longitudinal study	N = 74 undergraduate students CG: 37 EG: 37	CG: Traditional teaching EG: Active learning using an AR mobile application	Two weeks Content: Anatomy of spine, and spine pathologies	Post measurement Structured questionnaire about level of understanding and engagement.	Significant results were found for the EG group on level of understanding and engagement.	6/9
Hartstein et al. 2022. USA [38]	Randomised controlled study	N = 59 graduate students (1st year)	CG: Traditional standardised patient instruction EG: Immersive VR learning experience with Oculus Quest 2	One lesson Content: simulation of a patient encounter to enhance clinical decision-making skills	Pre-post measurement Clinical decision-making tool Metacognitive Awareness Inventory Diagnostic accuracy and efficiency Engagement Musculoskeletal objective structured clinical examination	Non-significant differences were found between groups for the clinical decision-making tool, metacognitive awareness inventory, diagnostic accuracy. Only the results for engagement are significant for the EG.	10/13

AR: Augmented reality; CG: control group; EG: experimental group; HMD: head-mounted display; N/A: not available; VR: virtual reality.

## Data Availability

The study did not report any primary data.
